# Releasing the Caged Phoenix: COVID-19 and the Rare Opportunity Afforded to Ontario's Teachers

**DOI:** 10.1177/10567879211015943

**Published:** 2022-07

**Authors:** Jaime Whitley, William T. Smale

**Affiliations:** 1 Otonabee College, School of Education and Professional Learning, 6515 Trent University, Peterborough, Canada

**Keywords:** COVID-19, education, pedagogy, teachers, curriculum, school administration

## Abstract

This paper addresses perceptions of how Ontario's response to the COVID-19 pandemica has changed the role of teachers. It explores the opportunity for Ontario's teachers to reframe their approach to curricula and pedagogy through their own personal leadership and to use the current pandemic landscape as a gateway rather than a barrier to growth. It chronicles the first wave of the pandemic response in Ontario's education sector, reveals windows of opportunity that have existed and continue to exist within current education policy, and explores scholarly work in the areas of *currere*, lived curriculum, localized curricula, and leadership in the spirit of learning and living.

## Introduction

In recent weeks, we received correspondence from a former student who shared the news that she was starting medical school in a week. Her message about the importance of community health care and her passion to give back to her small community by one day returning as a practicing physician was both heartwarming and inspiring.

As educators, we bask in these moments, when former students find their paths, share them with us, ultimately refreshing our sense of purpose. These are the students we are most proud of, it seems the ones who leave us and move on to greater things, the ones who make contributions to their fields and communities, and the ones who remember us fondly and come back from time to time to visit and share their stories. We are proud of them and sometimes covet their success as if we somehow crafted or molded them into something they were not before *we* graced them with *our* presence.

Reality check: the achievements were and should be all the students’. The truth is that the student described above, and others like her, came to us this way: primed receptacles for our teaching because they had already honed the discipline, work ethic, responsibility, resilience, optimism, and desire to learn which are necessary for success in school and in life. When we boast about our excellent education system, these are the images of students we hold in our minds, yet much like an iceberg, the story of education in Ontario runs far deeper, and sometimes darker, for so many students than what is revealed to the fair-weather observer.

There has been a great deal of talk, both formal and informal, about what COVID-19 has done *to* us in education. The purpose of this paper is to explore what COVID-19 has done *for* us. We argue that in many ways, COVID-19, and Ontario's response to it revealed to us our collective struggle to reach *all* students, a mandate that this and previous governments and union groups representing education workers across the province have striven for and even boasted about achieving for years. However, among “swinging pendula,” systemic improvement plans, economics, politics, collective bargaining, ever-changing leadership, and, quite simply perhaps, our own complacency as we cope with the aforementioned tensions, many of us have lost track of our *own* vision.

In terms that Sinek (2009) would use, many of us are missing a viable *why* to guide the *how* and *what* of our practice. Of all the complaints and concerns we heard over the past five months as we have been tasked with implementing learning at home, inundated with the “new” language of trauma-informed practice, and are now preparing to return to schools with ill-prepared students under grim environmental circumstances to ensure the health and safety of everyone, the greatest has been that we no longer know what the role of a *teacher* is. Our assessment and evaluation practices have been challenged, our concept of curriculum and what ought to be covered has been, for some, thrown out the window, and our overall integrity has been presumably trumped by the order that “No student [will be] adversely affected by school shutdowns,” loosely translated for most teachers, particularly in the secondary panel, as “Everyone gets a credit.”

Our individual and collective ability to anchor our practice in a viable *why* is a reflection of the personal leadership that is a greatly needed amongst many teachers in Ontario, and also of the genuine autonomy and responsibility afforded to us if we truly take up this station in the vitally important role of teaching other people's children. Therefore, what COVID-19 has done for us is this: it has revealed to us a silver lining—an opportunity to reframe our purpose and our work as teachers, starting with our *why*. It has invited us to dive deep into the icy waters to see the iceberg for what it truly is, and at the very least, it ought to compel us to improve. This period has been loosely described by many as the “Greatest Experiment in Public Education”: we hypothesize, then, that *if* we use this unique opportunity to reframe our approach to pedagogy and curriculum, starting with personal leadership, *then* we will be better situated to actually prepare *all* of our students for their own brand of success in the world unknown.

For years we have heard the pendulum-swinging metaphor that teachers use to mock the comings, goings, and returnings of old practices following popular, yet short-lived trends set by authors and consultants showcasing their innovative approaches to student engagement. This metaphor reinforces the ever so popular idea that “what is old is new again,” an idea which lends credence to the notion that “that which is old never needed to be changed in the first place.” Many things are wrong with our use of the pendulum as a metaphor in education, the least of which is that its center is a fixed, rigid point, and although it pivots, is essentially immovable. It is clearly time to refocus, to unhinge the pendulum, and to make room for a new metaphor, one that welcomes a regeneration of perception and perspective without rewriting the policy playbooks or investing millions of taxpayer dollars to wipe the slate clean and start from scratch.

Just as the phoenix emerges from its ashes to be born again, assuming the same structure it left behind, so too can we, Ontario's teachers, emerge from this great experiment, take on our role with fresh eyes, open minds, and young hearts, and embrace the opportunity for regenesis within ourselves and in our practice. In the process, hopefully, we can spread our wings and take our place as custodians of change in education.

## The COVID-19 Landscape: A Retrospective to Now

In an April 9, 2020 article immediately following the release of the Phase 2 Emergency Remote “Learning at Home” guidelines by the Ontario Ministry of Education (OME), Beyhan Farhadi, a PhD and Toronto District School Board educator, brought some glaring inequities in Ontario's education sector into the limelight. In this article, Farhadi responded to Education Minister Stephen Lecce's declaration that “school is back in session” with the simple question of “for whom?” (Farhadi, 2020, para. 3). This question is not lost in the context of equity: Farhadi identified access to devices, reliable internet connection, inequitable caregiver support, and a general “resource gap” as key concerns emerging from this learning model, taking note as well of the leverage offered to individual district school boards to take varied approaches to program delivery, leaving evaluation expectations, as well as prescribed hours of teacher-led learning and asynchronous versus synchronous work, unclear (Farhadi, 2020).

Farhadi's emphasis on the technological inequities was consistent with commonplace educator concerns: “while we envision technology as a means of democratizing learning, in practice, particularly while done at home, it is accessed by the highest-achieving students,” (para. 4) or at least, in my experience, the most driven and self-directed students (Farhadi, 2020). Farhadi used this argument to justify the discontinued practice of assigning grades and hard deadlines, particularly in a time of instability and unpredictability. To further this point, Farhadi (2020) wrote: “To roll out what has been a specialized program [e-Learning] serving a minority of students to the majority of students in an emergency—sets up expectations against which we are primed to fail” (Farhadi, 2020, para. 5).

At the same time, Farhadi (2020) addressed inequities students faced at home: “[The] provincial ‘Learn at Home’ approach draws not only on a fantasy of eagerly connected students with ample resources, but also on a fantasy of home free from conflict and space constraints, supported by caregivers who can and will provide structure, motivation, and mediate learning between the teacher and their child” (Farhadi, 2020, para. 6). In doing so, Farhadi exposed the reality that our students are often living in “precarious conditions” (para. 6), at times forcing them to work in essential services as contributors to the household, faced with food and shelter insecurities, emotional barriers, familial tensions, and overburdened parents.

Farhadi offered criticism of Stephen Lecce's declaration to parents that their children will require “academic discipline” and “commitment” (para. 7) to experience growth and achievement, assuming that Ontario students are “up to the task,” (para. 7) an indeed fantastical image of the Ontario student which looks a lot like our pre-med superstar, neglecting to take into consideration the 25% of Ontario students who require special education services and supports, as well as the countless others who require face-to-face structures that are not always available at home (Farhadi, 2020). Farhadi's article sought in many ways to praise district school boards for their commitments to student success, describing the efforts made in individual districts and their respective schools as “the equalizing force,” “moving mountains” for vulnerable families, ensuring that they have access to much-needed support (Farhadi, 2020, para. 6).

In an attempt to make the “Learning at Home” model equitable, Farhadi proposed renewed approaches to teaching, approaches which some educators might argue raise further questions and concerns in terms of the evolving role of the teacher. Perhaps the most alarming to some was the suggestion that Ontario teachers adopt a “trauma-informed approach,” which places well-being over behavioral compliance, supports connectivity, reduces stress, provides hope and grounding during the chaos, and focuses on “messaging that encourages routine and connection [as] more important than enforcing arbitrary measures of academic engagement” (Farhadi, 2020, para. 10).

Farhadi continued suggesting mindsets that teachers ought to take when approaching phase 2: the imperative that we strive to maintain social proximity virtually to ensure that students feel connected “even if they are not formally engaged in learning tasks,” and that we avoid applying the logic of traditional face-to-face classes online (Farhadi, 2020, para. 11). Here, Farhadi urged that for accessibility and equitability, “mass delivered remote instruction must be asynchronous, reproducible physically, and supplemented by optional recorded audio conferences or phone calls” (para. 12), asserting that “we cannot compel students online, if online learning doesn't work for everyone” (para. 12) and that “we cannot continue assigning grades if students are unable to demonstrate their learning” (Farhadi, 2020, para. 12). For many educators, particularly those Simon Sinek would call the “late majority” and “ laggards,” in reference to the Law of Diffusion of Innovation, these suggestions call for a radical shift in philosophy and practice, and are challenging, to say the least (Sinek, 2009, p. 116).

In a poignant and inspired conclusion, Farhadi (2020) called for reflection, study, and deliberation on the current landscape in education to draw attention to the inequities which have been “exacerbated during times of crisis” (para. 13), so that we can ultimately understand and better respond to our most vulnerable students (para. 13). Farhadi (2020) also cautioned that we must not normalize emergency measures as a means of rationalizing policy that “inevitably leaves students behind” (Farhadi, 2020, para. 13).

When we fast-forwarded to the 2020–2021 school year, here is what we found: in general, we survived, notwithstanding unprecedented fatigue, professional disorientation, and “Zoom” overload. Students “graduated,” so to speak, some were found, others were lost—but that has always been the real story of education in Ontario, Canada. The truth is, other than operational logistics and many more credits granted than were perhaps expected, the overall outcome was not much different than it had been before COVID-19 marched across our schoolyards, only now, we have an ethical and political agenda to look more closely at our education system.

On June 19, 2020, the OME released the much anticipated “Approach to Reopening Schools” for the 2020–2021 school year, giving, as a precedent, significant leverage to boards to make local decisions, or rather to “be prepared to” make local decisions given the continued uncertainties around public health, within the set parameters of the document. A frontline expectation of this document was that boards be “prepared with their plan for the upcoming school year by August 4” (para. 5) in the areas of voluntary school attendance, school organization and timetabling, and ensuring academic success (Ministry of Education, 2020a).

As to voluntary school attendance, the OME stated that attendance would be at the discretion of parents for the 2020–2021 school year and that boards should be prepared to offer remote education when parents request it. A number of options for boards to consider for continued remote at-home delivery were provided with the expectation that boards establish “direct synchronous instruction [for students] with their teacher on a regular basis” (para. 44). The report goes on to note that “synchronous learning can be used as part of whole class instruction, in smaller groups of students, and/or in a one-on-one context” (Ministry of Education, 2020a). One breath of fresh air was the intended return to assessment, evaluation, and reporting practices “as usual,” nullifying emergency local memoranda that trumped provincial policy and left teachers’ heads spinning in the spring.

Turning to school organization and timetabling, boards were asked to be prepared for programming within normal days with enhanced public health protocols, as well as modified instructional days based on smaller class sizes, cohorting, and an alternative day or week delivery, in addition to the aforementioned at-home learning. The document stated that “school boards may need to be nimble and adopt one or more of these forms of delivery through the school year” (para. 15) and drew on direction from public health advice regarding “distancing” and “cohorting,” which refers to minimizing the number of students and teachers that any individual comes into contact with.

“Approach to Reopening Schools” offers boards suggestions for each, most of which involve timetable adaptations keeping students in one class together with one single teacher throughout the day where possible. Recognizing the challenges cohorts pose in secondary schools, the document stated that “the ministry anticipates that schools and boards will identify a range of timetabling and delivery approaches that reflect the goals of distancing and cohorting and is willing to review and discuss all reasonable adaptations” (Ministry of Education, 2020a, para. 35).

Refresher learning for the purpose of ensuring academic success was another hot topic, specifically in secondary settings where schools operate by semesters, and sought, without real direction, to level the playing field for students who had missed opportunities to access prerequisite curricula in the spring. While this missed opportunity is of grave concern for secondary school teachers, the document merely pointed out that boards must ensure that “content review for students is integrated throughout the school year at key instructional times to ensure students have fundamental building blocks before each new unit” (Ministry of Education, 2020a, para. 55). For many teachers, this requirement does not answer concerns about potentially three and half months of missed instructional time, given the rate of student opt-outs following the March 13, 2020 threshold for maintaining passing grades.

“Approaches to Reopening Schools” was highly unpopular, as outlined in a June 23, 2020 article in *The Toronto Star*. Of specific note was the OME's propensity for leaving the “heavy lifting” (para. 8) of decision making to District School Boards and the argument that this “patchwork” (para. 8) approach across the province's 72 school boards may not serve the best interest of students (Star Editorial Board, 2020). The article did acknowledge the need for flexibility across broad geographical, and resulting demographic, landscapes and the public health concerns unique to each, yet points a harsh finger at the deflection of responsibility from the OME to local officials with such vague direction but pronounced expectation outlined in the document (Star Editorial Board, 2020).

On July 30, 2020, the Ministry of Education provided more details for Ontario educators and families, including further direction for local boards, on “reopening schools,” which seemed to provide a complete distraction from the inequities revealed in the spring with widespread fear of a potential public health nightmare. The first order of business in this document was to determine “designated” and “nondesignated” school boards based on risk assessments by local public health offices. Secondary schools in those districts deemed “designated,” mostly those surrounding the Greater Toronto Area, would open by following adapted, blended models, with students attending on-site 50% of the time and class sizes reduced to 15 to support previously established “distancing” protocols. “Nondesignated” boards would return to regular, fully operational on-site instruction with specific health and safety measures in place.

It is interesting that a reduction of class sizes was not required in “nondesignated” schools. In fact, the document stated that “secondary schools in these boards typically have fewer students, fewer schools and smaller class sizes” (Ministry of Education, 2020c). While public health officials would be involved in the handling of suspected and confirmed cases of COVID-19 in schools, including the decisions for resulting school closures, the document also outlined the responsibilities of staff and families to self-monitor and respond to reports of COVID-19 within public health guidelines (Ministry of Education, 2020c).

A more comprehensive “Guide to Reopening Ontario's Schools,” published by the OME on July 30, 2020, detailed the nature of cohorts and distancing relating to timetables in secondary schools, leading to a cacophony of boards’ public releases of plans, public redactions of plans, and public revisions of plans that would follow in the weeks approaching school and test the patience and confidence of Ontario teachers and families. Of significance was the statement that “a secondary student should be limited to approximately 100 student contacts” (para. 50) and that “boards are also encouraged to keep in-person cohorts to two classes, or with their grade, depending on the size of their high school” (Ministry of Education, 2020b).

In this newer guide, the term “quadmester,” was introduced as an alternative to the typical semester where students would take four courses per day. In the “quadmester” students would take two courses per day or in alternating weeks to reduce contacts for each individual, completing the first two courses at the typical mid-term period, at which point they would begin their remaining two courses.

Following this guideline, many Ontario boards publicly released plans to do just that toward the end of August, but, recognizing soon after that those conditions would still challenge the 100-student contact threshold, many canceled their decisions and opted instead for an unprecedented “octomester,” in which each course would be delivered all day in a five-week block, demanding a co-teaching model be employed to maintain compliance with established collective agreement language regarding teacher workload. Many boards released this information to the public in devastatingly short order, in an almost domino-like response to neighboring districts, during the last week of August as teachers and families were in the midst of last-minute preparations for back-to-school.

In regard to social distancing, the Ministry of Education (2020b) stated that: “as much distancing as possible between students, between students and staff and between staff members should always be promoted…classroom sizes in Ontario vary…but schools are encouraged to remove unnecessary furniture and place desks with as much distancing as possible…” (para. 81). This guideline, without the mandated measure of reduced class sizes for “nondesignated” boards, left teachers with a pervasive fear of being unable to safely accommodate upwards of 30 learners in one space at one time.

As another last-minute measure, school boards were tasked with polling families on whether they intended to send their children to school or to opt for voluntary remote learning, the result of which was another unprecedented move by most Ontario boards to open brand new K-12 “virtual schools,” supported by August 13, 2020, *Policy/Program Memorandum 164* outlining requirements for remote learning. As an accommodation for this late option, the OME offered a two-week grace period for the opening of regular and virtual schools, also a last-minute resort, resulting in many districts opting for an official start date of September 14, 2020, with an additional staggered entry for students, opening fully to all children on September 18, 2020.

As we write this very timely piece, we are bombarded with messages of fear, anxiety, and at the very least, apprehension. While concerns for the health and well-being of our students and our families are by no means unwarranted, taken against the backdrop of the potential that our current situation has afforded us, we maintain that it is far more prudent for us to listen for the few but powerful voices acting as beacons of hope; those who do believe that *we are* up to this task. The issue is one of efficacy, resilience, and empowerment which will be explored in the remainder of this paper. But first, a realization.

## A Lost Decade of Authentic Evaluation and Well-Being Awareness in Education Policy

The current landscape in education, the COVID-19 landscape, has introduced a new language, or familiar language in new contexts, for educators, much of which has contributed to their individual and collective disorientation around the changing role of the teacher. OME directed and system reinforced expectations for evaluation and reporting to reflect only the achievement of “overall expectations” and Farhadi's call for “trauma-informed practices” seem to be specific game-changers that have garnered harsh criticism amongst educators, particularly amid feelings of lost integrity, yet, to put it bluntly, these concepts are not new in Ontario's education policy.

In 2010, the OME released *Growing Success*, designed to guide the assessment, evaluation, and reporting practices of K-12 teachers. *Growing Success*, as our current policy on assessment, evaluation, and reporting, seeks specifically to offer opportunities for individual students to “achieve success according to [their] own interests, abilities, and goals” by “allow[ing] students to learn in ways that suit them best” (Ministry of Education, 2010, p. 1). This document reinforces the importance of the educator's professional judgment in providing “creative and judicious” differentiation in instruction and assessment and seeks to recognize the varied local needs from district to district, providing guidelines to foster such flexibility (p. 2). The touchstone of this document is the call for educators to adopt “new approaches to assessment [which] provide both opportunities and challenges to all educators, for the benefit of all students” (p. 3).

*Growing Success* outlines seven fundamental principles that lead to the improvement of practice for all students; three are of specific note: teachers must use practices and procedures that “are fair, transparent and equitable for all students,” “are carefully planned to relate to the curriculum expectations and learning goals, and, as much as possible, to the interests, learning styles and preferences, needs, and experiences of all students,” and “are ongoing, varied in nature, and administered over a period of time to provide multiple opportunities for students to demonstrate their full range of their learning” (p. 6).

On curricula expectations, *Growing Success* clearly outlined that from Grades 1 to 12, “all curriculum expectations must be accounted for in instruction and assessment, but evaluation [the judgement involved in assigning a percentage grade] focuses on students’ achievement of the overall expectations…[which] are broad in nature” (p. 38). *Growing Success* also addressed the teacher's role in “creating environments in which all students feel valued and confident and have the courage to make mistakes” (p. 8). The document stated that “in their important professional role, teachers show students that they care about them, and model a love of learning that can deeply influence their lives” (p. 8).

A careful examination of the front matter of *The Ontario Curriculum* from 2013 onward not only will reveal an echo of the evaluation policy surrounding the “overall expectations” outlined in *Growing Success*, but also an evolution of language to reflect a further focus on student health and well-being. In a new section of front matter entitled “Supporting Students’ Well-Being and Ability to Learn,” post-2013 curriculum documents require teachers to support and enable “all students to reach their full potential,” recognizing that health and well-being are contributing factors to learning in all disciplines and also that the educator's role in “creating, fostering, and sustaining a learning environment that is healthy, caring, safe, inclusive, and accepting” is a proactive measure to ensure health and well-being (Ministry of Education, 2016a, p. 3).

The research-based section of the front matter identifies the “determinants of health” that have been shown to affect a person's overall state of well-being, including “income, education and literacy, gender and culture, physical and social environment, personal health practices and coping skills, and availability of health services,” all influencing whether a person is physically healthy and will have the “physical, social, and personal resources needed to cope and to identify and achieve personal aspirations,” all having an impact on a student's ability to learn and his or her subsequent performance, and therefore critical to a student's success in school (p. 4).

Embedded within this front matter is also a list of research-based teachers’ resources and how they can be used to enhance the cognitive, emotional, social, and physical development of their students. Mental health is also explicitly noted as it relates to all components of a student’s development. Specifically, the document stated that “mental health is much more than the absence of mental illness” and identifies well-being as being “influenced not only by the absence of problems and risks but by the presence of factors that contribute to healthy growth and development” (p. 5).

Growing Success went on to describe the role of teachers in nurturing and supporting students’ “strengths and assets” to promote positive mental health, identify those who need additional support, and influence their overall well-being through the implementation of meaningful instructional approaches and the development of a supportive classroom environment for all (p. 5). Thus, provincial officials in Ontario continued their efforts to promote well-being, of which, mental health is one of four key pillars, as a fundamental component of education through the development of the 2016 *Well-Being Strategy for Education* to guide future policy and educator practice (Ministry of Education, 2016b).

Reminders for educators to evaluate only the overall expectations of their respective curriculum during the spring shutdown, and the resulting frustration of many teachers, struggling to navigate that “new” and “emergency” direction, is an indication that we need to do a much better job of understanding and enacting policy that has been in place for a decade. Farhadi's call for educators to take up “trauma-informed” approaches in our current practice is another indication that we have yet to embrace the direction outlined in curriculum policy since 2013. While a “trauma-informed approach” is not directly mentioned in these documents, its absence is merely a matter of semantics, for it is synonymous with practices that support overall mental health and well-being. These two areas have revealed a professional blind spot blocking our view of the permission we so urgently seek to employ our desired autonomy in decision-making and judgment within the classroom in balance with our critical responsibility for supporting the whole student.

## Starting with Why: Discovering our Personal Leadership as Said's Amateur Intellectuals

Personal Leadership Resources have been a touchstone to the conversation among education leaders in Ontario since the Institute for Education Leadership published its 2013 revision of the *Ontario Leadership Framework* (OLF). While this is an important revision, the inclusion of Personal Leadership Resources reads as a mere addendum to the existing framework, outlining key personal resources that leaders should possess to fulfill the complex responsibilities of various leadership roles, including cognitive resources, social resources, and psychological resources.

A critical look at the *OLF* reveals an overall absence of emphasis on “leader character,” focusing instead on the belief that successes in leadership roles emerge from that which can be learned. Still, the Personal Leadership Resources do echo, and in many ways, provide loose guidelines for the quality of person that systems should choose as leaders, indicating that both innate *and* developing character traits have much to do with success, if not more than that which can be learned through professional reading and continuing education course work. It is interesting to note that Personal Leadership Resources outlined in the *OLF* seem to be systemically reserved for those seeking formal leadership roles and used exclusively in frameworks for promotion.

One critical piece of learning has been our realization that “personal leadership” spans far beyond the professional realm of current and aspiring leaders, as the *OLF* might suggest; rather, personal leadership also involves our habits of mind, perspectives, and perceptions, and also our ways of being. Personal leadership forges our ever-evolving vision and determines our *why* in everything that we do in our lives, far beyond, but also within, our work. It has the potential to be our lifeblood as we connect with stories, engage in studies, nurture a marriage, raise our children, foster relationships, influence and inspire others, and yes, ultimately strive for happiness, fulfillment, and excellence in our life and work. Thus, for the purpose of this paper, “personal leadership” and “leader character” are intended to apply to the whole person, not simply someone seeking promotion to the formal role of leader in their work, something that we believe has been drastically overlooked and undernurtured by educators, having an equally drastic impact on our individual and collective efficacy.

In *Start with Why: How Great Leaders Inspire Everyone to Take Action* (2009), Simon Sinek provided a fresh look at “the golden circle,” a framework that has existed in various cultures throughout history, lending itself to lasting success, sustainability, innovation, and flexibility (p. 50). Based on the golden ratio, the golden circle seeks to find order and predictability in human behavior and has clear applications to biology, particularly connected to the functionality of the limbic brain (p. 56). In essence, the golden circle “helps us understand why we do what we do…provid[ing] compelling evidence of how much more we can achieve if we remind ourselves to start everything we do by first asking why” (p. 38). Sinek drew primarily on business models to illustrate how “starting with why,” or using an “inside out,” “why, how, what” approach distinguishes one company from another; he also recognized that this model has multiple applications in multiple fields (p. 38).

We often wonder how many of our colleagues would answer the question “Why do you teach?” We would hazard a guess that some commonplace answers might be “Because it’s a great job,” “We get weekends and summers off, great benefits, a pension,” and “We like the kids.” Moreover, there is nothing wrong with these answers. However, what if we were to dig deeper, to insist upon a substantial *why* that exists beyond the superficial benefits of unionized government work, and get to the heart of the matter? At this moment, we are reminded of our graduate studies peers just beginning their careers as educators: the millennial of our profession, not yet disappointed, let down, or left with their spirits crushed by the day-to-day challenges of the profession; bright-eyed eager-beavers full of love, empathy and, most of all, hope, who might naturally dig deeper to find and shamelessly share their *why* as a passion for the raw talent of youth and the opportunity to somehow shape the future.

The unbridled confidence and dedication of new teachers, often trivialized by seasoned educators, distinguishes and often stereotypes this group from the rest of us, namely because they are willing to declare themselves publicly as such. We find ourselves wondering if, beneath their seemingly overzealous facades, these colleagues of ours are on to something, if this vulnerability they seem to hone as a superpower *is* actually a superpower, one that the rest of us have yet to discover. We are reminded of one such millennial who relentlessly pushed us o learn something new and unsettling, and handled our resistance with this simple statement of commitment: “We’re doing this, and it's going to be okay because I’m going to help you.” Now, that is character.

How do the rest of us crack the surface and (re)discover our *why* beyond the job itself? On our own personal, professional, and scholarly journeys, we have come to discover our own *why*, or at least my *why* as it exists today, which has been the most valuable learning for me and has become the building block for my own personal leadership. Inspired by the work of Sears and Cairns (2010), we have come to understand that “real learning is not just acquisition, it is transformation; we are changed by it” (Sears & Cairns, 2010, p. 65). Thus, we now understand that we teach because we believe in the transformative potential of education, a potential that ceases to exist without hope.

Transformation in education is not limited to the experience of the youth in our charge. In an unexpected opportunity embedded within the context of curriculum theory, we were introduced to Kanu and Glor’s (2006) article entitled “Currere to the rescue? Teachers as amateur intellectuals in a knowledge society,” in which they explore Pinar’s (1975) autobiographical method of currere as a beginning point for the transformation of teachers into what Edward Said called “amateur intellectuals”:

The intellectual today ought to be an amateur who considers that to be a thinking and concerned member of society one is entitled to raise moral issues at the heart of even the most technical and professional activity…The intellectual's spirit as an amateur can enter into something more lively and radical; instead of doing what one is supposed to do, one can ask why one does it, who benefits from it, and how it can reconnect with a personal project and original thoughts. (Said, as cited in Kanu & Glor, p. 103)

Essentially, in foregrounding the relationship between one's life history or narrative and one's teaching practice, Pinar's currere provides opportunities for teachers to “theorize particular moments in [their] educational history, to dialogue with those moments, and examine possibilities for change” (p. 104). For Pinar, then, currere is an existential experience that we engage in “so that we may see more of [the institutional structures] and see [them] more clearly” (p. 104). Kanu and Glor brought the act and art of storytelling to the forefront, and if there is something that all seasoned teachers have in common, it is our propensity to tell and retell stories: chronicles of the people and places we have encountered throughout our careers that somehow become a measure of who we are. As time passes, often too they become measures of who we were. Memories are both inspiring and haunting.

We are overwhelmed at this moment with recollections of retirement addresses that we have heard over the years, riddled with these stories, “the highlights” of one's career that we could all “write books about.” In the spirit of Said, as explored by Pinar, Kanu, and Glor, we would suggest that our stories are an appropriate and accessible starting point to transform ourselves as “amateur intellectuals,” in the process of discovering our own *why* as we begin our journey toward personal leadership. We genuinely believe that the potential to envision possible futures in education as we explore these stories become endless should we choose to engage in this work.

## Reshaping our How: Theory as Liberation and Embracing the Tensions of *Lived* Curriculum

For some odd reason, despite holding a minimum of two postsecondary degrees, with all of their “rights and privileges,” educators often choose to end their tenure as scholars on employment. While they usually engage in professional learning, this rarely includes current scholarship and theory in education. Should we seek to enact our *why*, to transform ourselves into “amateur intellectuals,” we must reconsider this engagement. Hooks (1994) identified theory as a “location for healing” which we must enter into if we are to imagine possible futures (Hooks, 1994, p. 59).

“[T]heory is not inherently healing, liberatory, or revolutionary”; Hooks wrote, “it fulfills this function only when we ask that it do so and direct our theorizing toward this end” (p. 61). Hooks took on anti-intellectualism in education, stating that “by reinforcing the idea that there is a split between theory and practice or by creating such a split, both groups deny the power of liberatory education for critical consciousness” (p. 69).

Intellectualism, in this case, is not designated by an undergraduate degree or two as the benchmark to pursue a career in education; it is, in fact, designated by our willingness to access theory, engage with it, and be as reflective and reflexive as Pinar, Kanu, and Glor compel us to be: to become Said's “amateur intellectuals.” If we become hooks’ anti-intellectuals, then we too remain unable to fully capture our own essence and the essence of our students and our work. In the spirit of Freire, Hooks wrote from a place of hope. Her epigraph, which we like to think is an homage to Freire, says it all: “…to begin always anew, to make, to reconstruct, and not to spoil, to refuse to bureaucratize the mind, to understand and to live life as a process—live to become….”

Aoki (2009) reframed the concept of “metonymy” in the context of education as the “this OR that” (p. 180) polarized spaces in which many educators live and which limit our engagement with the ever-so-important tensions from which growth emerges and hope exists. Aoki asserted that we choose to live within these spaces because we fear the space between, the “ambivalent, uncertain and ambiguous” site which is actually a place of vibrant “doubling of language” where things can indeed exist as “this AND that” (p. 180).

Aoki's powerful analogy of the violin string is a perfect illustration of the vibrance of this space in which we ought to dwell: “to be alive is to be appropriately tensioned and to be tensionless, like a limp violin string, is to be dead.” In teaching, as in life, these metonymics exist in the stories we hear, tell, and retell. When examined closely, they serve as an affirmation that when we retreat to these polarized sites of safety, we are in fact playing with a metaphorical death, or are, at the very least, paralyzed in the face of the possibilities that tensions afford.

For Aoki (2009), hope in education is manifested in “curriculum-as-lived.” Aoki called for the “legitimation of curriculum-as-lived,” “a call to recognize the textured site of the lived tension—so often ambiguous, uncertain, and difficult—and a call for struggle in tension but nevertheless a generative site of possibilities and hope” (p. 180). For Aoki, as we engage in curriculum-as-lived, we begin to reconsider images of the self and the other and to rethink curriculum and pedagogy in this spirit. To illustrate this point, Aoki compelled us to consider the Taoist teaching of Roshin, who said, “Humanity's greatest delusion is that I am here and you are there” (p. 181). Hope emerges when we are open to new images of the self and the other, and likewise, new possibilities emerge when we are open to the relationships between them.

## Re-Imagining our What: A Localized Approach to Education

Every so often, our response to the question of “What do we teach?” is embedded in a subject or curriculum document: “I teach history,” “I teach English,” “I teach calculus,” or “I teach physics”; yet, occasionally, someone, perhaps one of our millennial friends, cleverly replies, “I don't teach curriculum, I teach students.” Once again, for poststructuralists in the field of curriculum theory, this new breed of teacher might just be on to something. Schwab (1973) and Ladson-Billings (2014) lend credence to this clever notion by their attempt to advocate for a localized curriculum. Before we proceed, it is important to note that the dominant language in Ontario's education policy isolates the term “locally developed” as a designation for a curriculum aligned with that which was formerly known as the “basic” pathway, language lending itself to vocational work, and in stark contrast to academic or scholarly work. Moving forward, we must disassociate ourselves from this designation to re-imagine the possibilities afforded to us should we take up a localized approach to education for *all* students.

Schwab (1973) maintained, “decisions made away from the classroom and the school would not capture local conditions” (Schwab, 1973, p. 79). For Schwab, abstractions about curricula were to be avoided because “real acts, real teachers, real children are richer and different from their theoretical representations” (pp. 79–80). Curricula, then, are constituted by far more than regulatory documents outlining expectations and objectives; curriculum instead, is contextual, taking not only the subject matter, but also the students and the milieu or classroom climates into intentional consideration, something we believe that careful attention to *Growing Success*, in synergy with curricular front matter, reveal.

For Schwab, embracing the local, then, is paramount to *doing* curriculum; neglecting to do so is to discredit the agency of the people, students, and teachers, as they are situated in a particular time and place. A local agency is also of importance when we consider Ladson-Billings’ (2014) culturally sustaining pedagogy. Culturally sustaining pedagogy seeks to push the boundaries of culturally relevant pedagogy in that it rejects superficial trends in education, forces questions of equity and justice, and pushes teachers and students to consider global identities. These goals cannot be accomplished, however, unless we first consider how *our own* curriculum, in the spirit of Schwab, brings about a sustainability of culture. Ladson-Billings (2014) wrote:

If we ever get to a place of complete certainty and assurance about our practice we will stop growing. If we stop growing we will die and, more importantly, our students will wither and die in our presence…students and teachers are vulnerable to classroom death; teachers who stop trying to reach all or who succumb to rules and regulations that are dehumanizing and deskilling…[are] functionaries of a system. (p. 77)

We think this warning is perhaps one of our greatest concerns emerging from the current landscape and conditions we find ourselves in. Fear, anxiety, and at the very least, apprehension over the changing role of the teacher during “COVID times” lend themselves to a very critical realization that we may very well be on a path to an imminent metaphorical death in education, bringing our students along with us. Aside from the few hopeful, individual visionaries who choose to forge on, we are at risk of becoming the proverbial frog, slowly boiling to death if we neglect to enact change in response to our new conditions. Therefore, here we are, finding ourselves at a crossroads, in a unique position to rapidly evolve or atrophy to death: to (re)discover ourselves as our own personal leaders with a vision and a viable *why*, to reshape our thinking, and to re-imagine possibilities for our life and our work.

## Reopening the Canon, Reframing Leadership, and Releasing the Phoenix

We are all, or at least likely were once, vaguely familiar with Freire and his writings from our pre-service teacher education. In *Pedagogy of the Oppressed*, Freire (1970) described libertarian education, the authentic humanist educator, and the transformative potential of people through an abandonment of the banking system of education in favor of problem-posing education. Appropriately for a visionary, Freire referred to this type of education as “prophetic,” or, in other words, “hopeful” (Freire, 1970, p. 84). Freire's vision was firmly grounded in his unwavering belief in people, human beings who are in a constant, transformational state of “becoming” (p. 84).

In education this vision calls for us to embrace the process of becoming, to engage in acts of both cognition and consciousness, acts of authentic thinking, and, ultimately, to solve the teacher–student contradiction that is the basis of the banking system—to change the story of education. Freire warned us of the well-meaning, “misguided” educators: “those who espouse the cause of liberty [yet] are themselves surrounded and influenced by the climate which generates the banking concept, and often do not perceive its true significance or dehumanizing power” (p. 70). In short, these are the educators we are at risk of becoming, albeit unintentionally if we are not already there.

As a true visionary, Freire, in his later work, offers us more tangible hope of acting out his vision. In *We Make the Road by Walking* (1990), he and Miles Horton explicitly addressed the nature of change in schools through candid dialogue. In response to Horton's question “How much have schools changed?” Freire interestingly replied *not that they have* changed, but that they *can be changed* “by a new generation of teachers, of educators who must be prepared, trained, and formed” (Horton & Freire, 1990, p. 220). Freire asserted that this act of formation is a “permanent process” of critically understanding what we do, an engagement in Freire's “dynamic present” so that we can be fearlessly revolutionary, disrupting cycles of oppression, perhaps represented through disengagement, by embracing an authentically humanist approach.

In *The Power of Reframing*, Bolman and Deal (1991) attributed organizational failure to a lack of imagination and courage among an organization’s leaders (p. 4). Perhaps the problem is more than such a lack; perhaps the problem is our limited capacity to recognize leadership within ourselves beyond the scope of a title or role. For Bolman and Deal, effective leaders are visionaries in action, and in essence, artists who “must use their artistry to articulate and communicate their vision so that others are able to see things differently” (p. 11). These authors wrote that “artistic leaders are essential in helping us to move beyond today's organizational forms to those that will release untapped individual energies and improve collective performance” (p. 18).

Bolman and Deal reminded us that we have the potential to fulfill Freire's vision, but we must first recognize our work as that humanist and liberating praxis, the demand for action, reflection, and learning. While we are not yet convinced that we are ready for Freire, we are convinced that there has never been a more compelling time to reopen the canon than in our new, very unprecedented context.

Many very real and tangible forces are working against Ontario's teachers in these “COVID times.” Forced with the adjustments to the “quadmester” and “octomester” blocks of intense learning with little guarantee of students’ prerequisite knowledge, the collegial demands of co-teaching, and the transience of students between on-site and remote learning in light of brand new virtual schools, Ontario's educators have been left in a position to either sink or swim—but seem destined to sink with the added weight of these burdens. Such forces have, most unfortunately, placed a dark and blinding veil over the opportunities afforded to educators in situ, in this current time, space, and social circumstance.

Coincidentally, these forces have also brought unexpected leaders of all kinds out of the woodwork as beacons of hope. Amidst the fear, anxiety, and apprehension are messages echoing the sentiments that “Together, we can do this” and “We can do hard things,” brilliant yet simple examples of efficacy, resilience, and empowerment that are brewing amongst our kind, perpetuated, of course, by individuals such as our millennial friend.

Addenda to these messages also include commitments to ourselves and to one another, our individual and collective well-being, as well as promises from our employers to be patient and kind as we navigate these deep and icy waters together. While doing so is no doubt easier said from the sidelines than done from the frontlines, these commitments are clearly an invitation to slow down, reflect and reconsider; in other words, an invitation to take up the work of Farhadi, Sinek, Kanu, Glor, Hooks, Aoki, Schwab, Ladson-Billings, and Bolman and deal in the spirit of Freire, to (re)discover ourselves and reframe our approach to life and work with the courage and confidence today's educational landscape demands, for both ourselves and our students.

## Conclusion

We recently come to a critical understanding of educators in Ontario, ourselves included: we do the best that we can…*as far as we know*. This realization covers manners of both virtue and sin: our seemingly limitless care and compassion for our students, our altruistic commitment in service to their well-being and achievement, our never-ending station as learners, and the dangers that exist should we neglect to both acknowledge and confront that which we do not yet know with a very real desire get better.

We have our work cut out for us. For education in Ontario to truly evolve, we must abandon the pendulum and release the phoenix; but to do so, we must have the conviction to embrace the responsibility that comes with the genuine autonomy that we seek. The invisible life preserver is within reach. It exists in current policy, in theory, and scholarship, and within ourselves should we choose to engage in this challenging work with fresh eyes, open minds, and young hearts. Only then, as we truly believe, will we reach *all* of our students and live up to our reputation as an excellent education system. *Ignited is the phoenix; the time for regenesis is now*.

## References

[bibr1-10567879211015943] Aoki T. (2009). Interview: Rethinking curriculum and pedagogy. Kappa Delta Pi Record, 35(4, Summer), 180–181. 10.1080/00228958.1999.10518454

[bibr2-10567879211015943] Bolman L. E. Deal T. E. (1991). Introduction: The power of reframing. Jossey Bass.

[bibr3-10567879211015943] Farhadi B. (2020). Educating Ontario students during COVID-19. https://broadbentinstitute.ca/educating_ontario_students_during_covid_19

[bibr4-10567879211015943] Freire P. (1970). Pedagogy of the oppressed. Continuum. https://commons.princeton.edu/inclusivepedagogy/wp-content/uploads/sites/17/2016/07/freire_pedagogy_of_the_opp resed_ch2-3.pdf

[bibr5-10567879211015943] Hooks B. (1994). Theory as liberatory practice. In teaching to transgress: Education as the practice of freedom (pp. 59–76). Routledge.

[bibr6-10567879211015943] Horton M. Freire P. (1990). Education and social change. In Bell B. Gaventa J. Peters J. (Eds.), We make the road by walking (pp. 199–226). Temple University Press. https://codkashacabka.files.wordpress.com/2013/07/we-make-the-road-by-walking-myles-and-paolo-freie-book.pdf

[bibr7-10567879211015943] Kanu Y. Glor M. (2006). Currere to the rescue? Teachers as amateur intellectuals in a knowledge society. Journal of the Canadian Association for Curriculum Studies, 4(2), 101–121.

[bibr8-10567879211015943] Ladson-Billings G. (2014). Culturally relevant pedagogy 2.0: A.K.A. the remix. Harvard Educational Review, 84(1), 74–84. 10.17763/haer.84.1.p2rj131485484751

[bibr9-10567879211015943] Ministry of Education. (2016a) Classical studies and languages Grades 9–12: The Ontario curriculum. http://www.edu.gov.on.ca/eng/curriculum/secondary/classiclang912curr.pdf

[bibr10-10567879211015943] Ministry of Education. (2016b). Ontario’s well-being strategy. https://collections.ola.org/mon/30005/334837.pdf-well being strategy 2016

[bibr11-10567879211015943] Ministry of Education. (2020a, June 19). Approach to reopening schools: 2020–2021. https://www.ontario.ca/page/approach-reopening-schools-2020-2021-school-year

[bibr12-10567879211015943] Ministry of Education. (2020b, July 30). Guide to reopening Ontario schools. https://www.ontario.ca/page/guide-reopening-ontarios-schools#list

[bibr13-10567879211015943] Ministry of Education. (2020c, July 30). Reopening schools. https://www.ontario.ca/page/covid-19-reopening-schools?gclid=EAIaIQobChMIhId7L7K6wIVi7bICh17rwAREAAYASAAEgKAOfD_BwE

[bibr14-10567879211015943] Ministry of Education. (2010). Growing success: Assessment, evaluation and reporting in Ontario schools. http://www.edu.gov.on.ca/eng/policyfunding/growsucces s.pdf

[bibr15-10567879211015943] Pinar W. (1975). The method of Currere. Washington, DC: American Educational Research Association.

[bibr16-10567879211015943] Schwab J. (1973). The practical 3: Translation into curriculum. The School Review 81(4, August), 501–522.

[bibr17-10567879211015943] Sears A. Cairns J. (2010). A good book, in theory: Making sense through inquiry. You are here: Mapping social relations. Broadview Press.

[bibr18-10567879211015943] Sinek S. (2009). Start with why: How great leaders inspire everyone to take action. Penguin Group.

[bibr19-10567879211015943] Star Editorial Board. (2020, June 23). Ontario’s back to school plan gets an F for incomplete. The Toronto Star. https://www.thestar.com/opinion/editorials/2020/06/23/ontarios-back-to-school-plan-gets-an-f-for-incomplete.html

[bibr20-10567879211015943] The Ontario Leadership Framework. (2013). The Ontario leadership framework: A school and system leader’s guide to putting Ontario's leadership framework into action (Revised ed.). The Institute for Education Leadership.

